# Application value of nucleic acid MALDI-TOF MS in mycobacterial species identification and drug resistance detection in *Mycobacterium tuberculosis*

**DOI:** 10.1128/spectrum.01545-24

**Published:** 2025-03-25

**Authors:** Xiaofang Liu, Honghong Niu, Donglin Guo, Huixia Gao, Lihong Wu, Jingyang Liu, Chunfeng Bai, Yuxi Li, Peilong Wang, Zhengfeng Zhou, Yuling Wang, Jianqin Liang, Wenping Gong

**Affiliations:** 1 Institute of Tuberculosis, Senior Department of Tuberculosis, The Eighth Medical Center of PLA General Hospital 12509, Beijing, China; 2 PLA General Hospital 104607, Beijing, China; 3 Hebei Key Laboratory of Immune Mechanism of Major Infectious Diseases and New Technology of Diagnosis and Treatment, The Fifth Hospital of Shijiazhuang, Shijiazhuang, China; Beijing Institute of Genomics, Chinese Academy of Sciences, Beijing, China

**Keywords:** nucleic acid, mass spectrometry, *Mycobacterium tuberculosis*, non-tuberculous mycobacteria, species identification, drug susceptibility

## Abstract

Tuberculosis (TB) and non-tuberculous mycobacteria (NTM) infections pose global health threats, requiring swift and accurate identification for effective treatment. This study aims to assess the ability of nucleic acid matrix-assisted laser desorption ionization time-of-flight mass spectrometry (MALDI-TOF MS) to rapidly identify *Mycobacterium tuberculosis* (MTB), NTM, and the drug resistance of MTB. A comparative analysis of 133 clinical samples was performed using acid-fast bacilli (AFB) staining, Lowenstein–Jensen (LJ) culture, GeneXpert, real-time PCR, and nucleic acid MALDI-TOF MS. The study focused on the diagnostic performance of nucleic acid MALDI-TOF MS in detecting MTB and NTM, as well as its accuracy in identifying the drug resistance profiles of MTB. The positive detection rate of nucleic acid MALDI-TOF MS for mycobacterium was 84.96%, which was significantly higher than that of AFB staining (29.32%). For NTM, nucleic acid MALDI-TOF MS had 89.29% sensitivity and 97.14% specificity, with an area under the curve (AUC) of 0.932, which was superior to other methods. The nucleic acid MALDI-TOF MS identified 28 NTM species, while real-time PCR identified only 12. Drug resistance detection showed concordance rates of 80% to 95% compared with drug sensitivity tests of LJ culture. Nucleic acid MALDI-TOF identified mutations, like KatG315 AGC-ACC for low-level isoniazid resistance, rpoB 531 TCG-TTG for high-level rifampicin resistance, and the InhA-15 C-T mutations, were also found in six isoniazid resistance cases and prothionamide resistance cases. Nucleic acid MALDI-TOF MS is a valuable diagnostic tool for the rapid and precise identification of mycobacterial species and the drug resistance profiles of MTB. With high sensitivity and specificity, it can guide the early initiation of effective anti-tuberculosis treatment in clinical settings.

**IMPORTANCE:**

Tuberculosis (TB) remains a critical global health challenge, exacerbated by the emergence of drug-resistant strains. Accurate, rapid diagnosis is imperative for effective treatment and control of TB. The ability to discern MTB from NTM is equally vital, as they demand distinct therapeutic approaches. This study underscores the significance of nucleic acid matrix-assisted laser desorption ionization time-of-flight mass spectrometry (MALDI-TOF MS) technology in providing a swift and precise diagnostic tool. Its high sensitivity and specificity in identifying mycobacterial species and their resistance profiles are paramount for guiding targeted anti-tuberculosis therapy. By potentially reducing the time to diagnosis and enabling personalized treatment plans, this technology could revolutionize TB management, ultimately mitigating its impact on public health.

## INTRODUCTION

TB is one of the major infectious diseases that pose a significant threat to human health (1, 2). According to the World Health Organization (WHO), there were approximately 10.8 million new TB cases globally in 2023, with nearly 1.25 million deaths and 400,000 new cases of multidrug-resistant (MDR)/rifampicin-resistant (RR) TB (3). China reported 741,000 new TB cases and around 29,000 new MDR/RR TB cases, making it one of the countries with the highest burden of TB and drug-resistant TB worldwide (3). Extrapulmonary TB accounts for 15% to 40% of all TB cases and has been a major focus in recent years due to its diagnostic challenges, potential for misdiagnosis, and the risk of functional or structural organ damage (4–7). The incidence of diseases caused by NTM is rising globally (8–11), and in many developed countries, NTM diseases have surpassed TB (12, 13). In China, the prevalence of NTM is also increasing annually (11, 14–16), with NTM lung disease being the most common (17). NTM lung disease shares clinical, radiological, and sputum smear acid-fast bacilli staining characteristics with pulmonary TB, and most NTMs exhibit inherent resistance or high resistance rates to commonly used anti-TB drugs (14, 16), posing a significant threat to human health and becoming a global public health concern. Accurate and rapid diagnosis and differentiation of TB and NTM diseases, as well as their drug resistance, are crucial for guiding early, precise, and effective clinical treatment.

Traditional TB diagnostic methods, such as MTB culture and drug susceptibility testing (DST), are considered the gold standard (18, 19). However, due to the slow growth of MTB, results typically take 4–8 weeks, significantly delaying timely treatment (20). The conventional method for culturing NTM begins with an initial screening using PNB differential medium to ascertain the presence of NTM. Following this, species-level identification is achieved through a composite assessment that includes growth kinetics, pigmentation, photoreactivity, and a series of sophisticated biochemical tests. However, the reliance on visual interpretation of these assays’ outcomes introduces subjectivity, potentially reducing the precision and reliability of the identification process. This variability can limit the clinical applicability of traditional culturing techniques, impacting the accurate diagnosis and effective management of NTM infections. Nucleic acid amplification-based detection methods (e.g., GeneXpert) can quickly detect MTB and rifampicin resistance but cannot comprehensively detect resistance to other anti-TB drugs. Furthermore, protein-based MALDI-TOF MS technology, which emerged in the 1990s, has been successfully applied for bacterial identification. However, most applications depend on the molecular mass differences of proteins and still require the culturing of mycobacteria, thereby limiting their rapid diagnostic capabilities. Consequently, nucleic acid MALDI-TOF MS technology, based on genetic sequence polymorphisms, was developed. Nucleic acid MALDI-TOF MS technology uses nucleic acids as biomarkers for bacterial identification (21, 22). The basic principle involves the specific amplification of multiple target gene fragments through multiplex polymerase chain reaction (PCR), followed by single base extension reactions at each site. The processed extension reaction products are mixed with the matrix required for mass spectrometry and run on a time-of-flight mass spectrometer, producing a unique fingerprint spectrum for each sample, which is then interpreted to obtain species and resistance information.

This study aims to analyze and compare the accuracy and reliability of nucleic acid MALDI-TOF MS with GeneXpert, real-time PCR, LJ culture, and DST in the identification of mycobacterial species and the detection of drug resistance in MTB. We evaluated the potential of nucleic acid MALDI-TOF MS for the rapid identification of mycobacterial species, including both MTB and NTM, and its application in detecting drug resistance in MTB. The focus was on enhancing diagnostic efficiency, reducing diagnostic turnaround times, and providing accurate drug resistance information to support personalized treatment. Nucleic acid MALDI-TOF MS can serve as a powerful complement to traditional culture and DST methods, facilitating the early initiation of effective and precise treatment by clinicians.

## MATERIALS AND METHODS

### Study subjects and design

**Fig 1:**
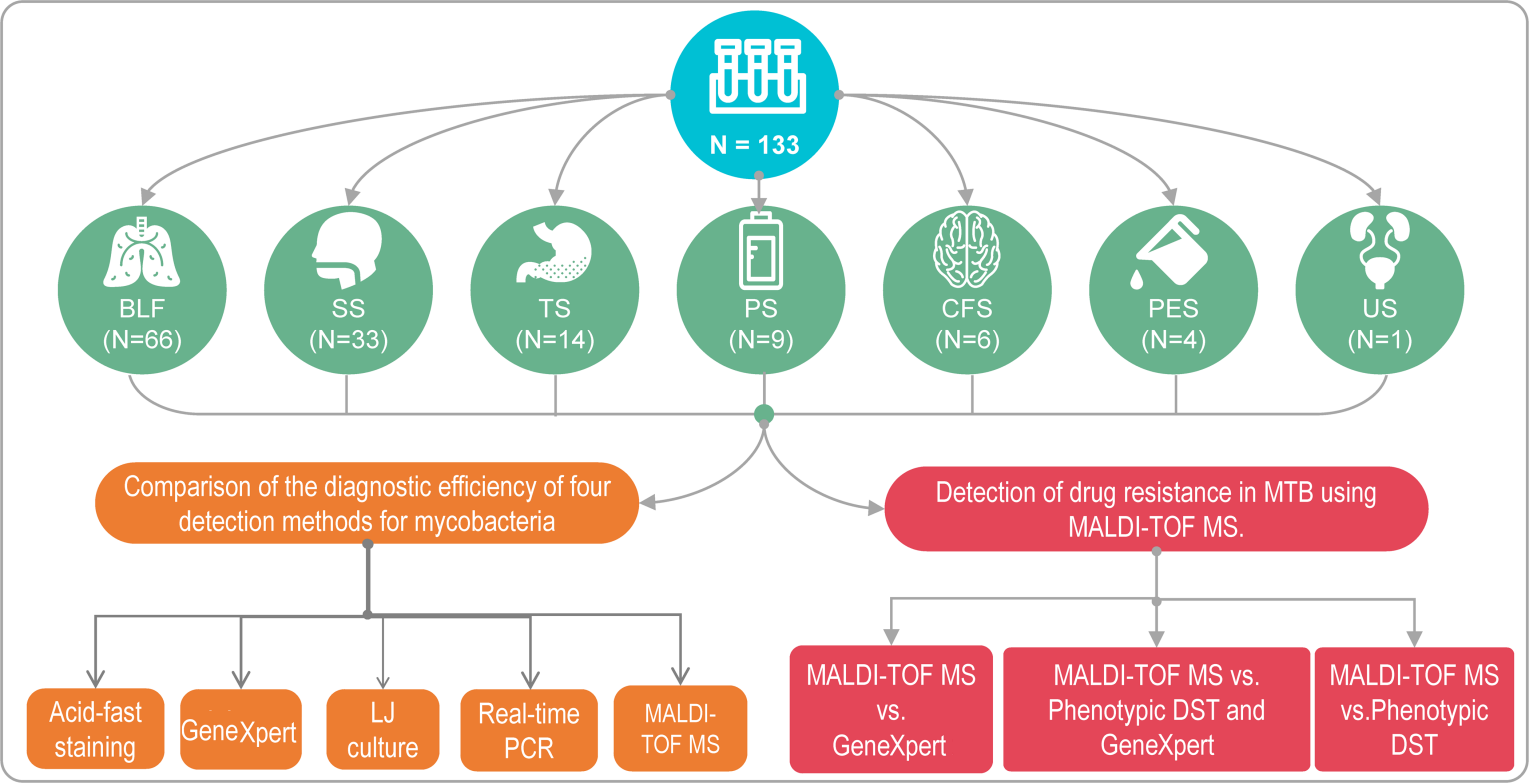
Schematic overview of the study design for evaluating the diagnostic performance of nucleic acid MALDI-TOF MS in mycobacterial identification and drug resistance profiling. This schematic diagram illustrates the comprehensive study design employed to assess the diagnostic capabilities of nucleic acid MALDI-TOF MS in the identification of MTB and NTM, as well as in profiling their drug resistance. The study encompasses a diverse set of 133 clinical samples obtained from the Eighth Medical Center of the PLA General Hospital, categorized into various types, such as bronchoalveolar lavage fluid (BLF), sputum specimens (SS), tissue specimens (TS), pus specimens (PS), cerebrospinal fluid specimens (CFS), pleural effusion specimens (PES), and urine specimens (US). The comparative analysis includes the evaluation of nucleic acid MALDI-TOF MS against four other diagnostic methods: acid-fast staining, GeneXpert, LJ culture, and real-time PCR, across different sample types and case numbers, as detailed in the figure. This study design provides a structured approach to understanding the efficacy and applicability of nucleic acid MALDI-TOF MS in clinical microbiology and resistance profiling.

This retrospective study collated 133 complete test results from inpatients in the Senior Department of Tuberculosis at the Eighth Medical Center of PLA General Hospital between December 2020 and April 2023. The demographic composition of the sample included 56 males and 77 females, with ages ranging from 12 to 86 years. The mean age was 43.56 years, with a standard deviation (SD) of 18.45 years, and the interquartile range (IQR) was 27.0 to 58.0. The clinical diagnoses among the patients were as follows: 97 cases of pulmonary or extra-pulmonary tuberculosis, 16 cases of NTM lung disease, 12 cases of NTM pulmonary disease (multiple species) and/or PTB, 1 case of bacterial meningitis, 1 case of lung adenocarcinoma, and 6 cases with indeterminate diagnoses. The samples included 66 bronchoalveolar lavage fluids (BALF), 33 sputum specimens, 14 tissue samples, 9 pus samples, 6 cerebrospinal fluids, 4 pleural effusions, and 1 urine sample. The study protocol was reviewed and approved by the Ethics Committee of the Eighth Medical Center of the PLA General Hospital. The study design is shown in Fig. 1.

### Diagnostic criteria

The diagnostic criteria refer to the Chinese Health Industry Standard “Diagnosis for pulmonary tuberculosis (WS 288–2017)” (23) and the Guidelines for the Diagnosis and Treatment of Non-tuberculous Mycobacterial Diseases (2020 Edition) (23, 24). TB diagnostic criteria: TB diagnosis can be made if there are systemic or localized symptoms of TB, radiological findings consistent with TB, and exclusion of other diseases, provided that the specimen is free of exogenous contamination and meets one of the following conditions:

1. Two specimens with positive acid-fast bacilli (AFB) smear;
2. One specimen with a positive AFB smear and radiological findings consistent with TB.
3. One specimen with a positive AFB smear and one specimen with positive mycobacterial culture, identified as *Mycobacterium tuberculosis* complex (MTBC);
4. One specimen with positive AFB smear and positive TB nucleic acid test.

NTM disease diagnostic criteria: NTM disease diagnosis can be made if there are systemic or localized symptoms of TB, radiological findings consistent with NTM disease, and exclusion of other diseases, provided that the specimen is free of exogenous contamination and meets one of the following conditions:

1. Positive NTM culture with two consistent isolates.
2. Histopathological evidence of mycobacterial disease (granulomatous inflammation or positive acid-fast staining) obtained via bronchoscopy or other biopsy methods, with positive NTM culture;
3. One specimen with positive AFB smear and positive NTM nucleic acid test.

### Detection methods

#### Ziehl–Neelsen acid-fast staining

In accordance with the Chinese Health Industry Standard WS288-2017 for the diagnosis of pulmonary tuberculosis, as detailed in Appendix B.2.6.2 (23), the procedure for Ziehl–Neelsen acid-fast staining is delineated as follows: (i) fixation; (ii) primary staining; (iii) rinsing with water; (iv) decolorization; (v) second rinse with water; (vi) counterstaining; (vii) third rinse with water. After rinsing with running water, the slide is dried and examined under a microscope, and the results are interpreted.

#### Lowenstein–Jensen culture, species identification, and drug susceptibility testing

According to the Chinese Health Industry Standard “Diagnosis for pulmonary tuberculosis (WS 288-2017)” (23), Appendix B.4, and “Standardized Procedures and Quality Assurance Manual for *Mycobacterium* Isolation and Culture” (25), samples were vortexed for 1–2 min and left at room temperature for 15 min before inoculating 0.1 mL of pretreated liquid onto two acid-modified Lowenstein–Jensen (LJ) slants. Cultures were incubated at 37°C and checked at days 3 and 7, and weekly thereafter for up to 8 weeks.

In accordance with the Standard Operating Procedure for the Isolation and Culture of Mycobacteria and Quality Assurance Manual, the preliminary species identification employs PNB medium supplemented with 500 µg/mL of p-nitrobenzoic acid (PNB). Strains that exhibit growth on PNB medium are classified as NTM. Conversely, colonies that proliferate on Löwenstein–Jensen medium in the absence of growth on PNB medium are identified as MTB. Fresh isolates of MTB are then inoculated into drug-containing media to assess colony growth, with the minimum inhibitory concentrations (MIC) being meticulously recorded.

According to the drug concentration and MIC value, it was divided into low-concentration drug-resistant strains, high-concentration drug-resistant strains, and sensitive strains. The DST of 10 drugs, including isoniazid (INH), rifampicin (RFP), ethambutol (EMB), streptomycin (SM), levofloxacin (LFX), kanamycin (KM), amikacin (AM), capreomycin (CAP), prothionamide (Pto), and para-aminosalicylic acid (PAS), was carried out. The reference doses for low-concentration resistance and high-concentration resistance of these 10 drugs were as follows: 1 and 10 µg/mL, 5 and 50 µg/mL, 1 and 10 µg/mL, 10 and 100 µg/mL, 50 and 250 µg/mL, 50 and 250 µg/mL, 1 and 10 µg/mL, 0.5 and 50 µg/mL, 10 and 100 µg/mL, 10 and 100 µg/mL, respectively. The strains identified as NTM were not further identified or tested for drug susceptibility.

#### GeneXpert MTB/RIF

GeneXpert MTB/RIF was performed according to the kit instructions (26–28). The kit used was GeneXpert MTB/RIF (Cepheid, Sunnyvale, CA, USA), detecting the rpoB gene and its mutations. Results were categorized as TB positive and rifampicin-resistant, TB positive and rifampicin-sensitive, or TB not detected. The GeneXpert MTB/RIF assay is employed on a variety of clinical specimens, each with specific volume requirements to account for the heterogeneity in bacterial load. The requisite volumes for processing are as follows: urine samples should exceed 50 mL, while pleural fluid and BALF should be in excess of 15 mL. Cerebrospinal fluid and purulent materials necessitate a minimum volume of 10 mL. All liquid specimens must undergo centrifugation at 12,000 rpm for 5 min to facilitate preparation. For fresh tissue samples, a minimum volume of 5 mm^3^ is mandated, and these samples must be homogenized prior to assay preparation. For sputum, 1 mL of sample was mixed with 2 mL of sample processing reagent and vortexed; for bronchoalveolar lavage fluid (BALF), cerebrospinal fluid (CSF), pleural and peritoneal fluid, pus, and urine, samples were centrifuged at 3,000×*g* for 15 min, the supernatant was discarded, and 1 mL of the pellet was mixed with 2 mL of sample processing reagent and vortexed. For tissue samples, they were cut into pieces, mixed with 2 mL of PBS buffer, and ground or homogenized to obtain a homogeneous suspension. Then, 0.7 mL of the suspension was mixed with an equal volume of sample processing reagent and vortexed. Subsequent steps were performed according to the GeneXpert MTB/RIF kit instructions.

#### Real-time PCR

The standardized procedures and interpretation of Real-time PCR followed previous studies (11, 29). Using a diagnostic kit (Capital Bio Technology, Beijing, China), MTB and NTM are identified by the PCR-fluorescent probe method, with specific targets referenced in the instructions. IS6110 was used as the target for MTBC detection, while the hsp65 gene served as the target for *Mycobacterium* species detection. Samples were mixed with an equal amount of 4% NaOH, incubated, and centrifuged. The supernatant was discarded, and normal saline was added, followed by another centrifugation. Nucleic acid extraction was performed, and the extract was heated before adding it to the PCR system. The amplification program included specific temperature and cycle settings. Detection was performed using fluorescein amidite (FAM) and VIC (a proprietary dye developed by Thermo Fisher Scientific) channels, with a Ct value <40 considered positive. MTB was identified when both IS6110 and hsp65 Ct values were below 40. NTM were identified if only the hsp65 Ct value was less than 40. A mixed infection of MTB and NTM was recognized when both Ct values were below 40, and the peaks were in close proximity.

#### Nucleic acid MALDI-TOF MS

Nucleic acid MALDI-TOF MS was conducted at Shanghai Conlight Medical Laboratory using the Conlight TB&DR assay (30–32). Key steps included sample liquefaction, nucleic acid extraction, PCR and extension reactions, desalting, and mass spectrometry analysis. The method can identify and distinguish eight subspecies of MTB complex and 40 species of NTM, detecting resistance to multiple anti-TB drugs based on 16 resistance genes and 29 resistance loci. The primary target gene for mycobacterium species identification using nucleic acid MALDI-TOF MS is the 16S rRNA gene. This gene is often combined with the *rpoB* gene, *gyrB* gene, and the 16S-23S rRNA internal transcribed spacer (ITS) to facilitate the accurate identification of *Mycobacterium* species and subspecies. For an in-depth understanding of the primers and probes utilized within the scope of this testing, one should consult the relevant national patents: CN113136446A, entitled “A Method, Primers, and Kit for Identifying Nontuberculous Mycobacteria and Detecting Drug Resistance Gene Mutations,” and CN113249502A, entitled “Related Genes, Method, Primers, and Kit for Identifying MTBC and Detecting Drug Resistance.” (33, 34) These patents elucidate the specific sequences of the primers and probes, which are pivotal for the accurate identification of NTM and MTBC, as well as for the detection of mutations associated with drug resistance in these mycobacterial species.

### Statistical analysis

#### Data collection and preprocessing

Clinical samples are systematically collected and processed in accordance with standardized protocols, ensuring the consistency and reliability of the data obtained. During the data cleaning phase, extraneous spaces are meticulously removed, and duplicate entries are eliminated to mitigate the impact of redundant information that could potentially skew the data quality. The test outcomes are encoded using binary values, where “1” indicates a positive result (MTB, NTM, or co-infection) and “0” indicates a negative result.

#### Confusion matrix and performance metrics

A confusion matrix was constructed by comparing detection results with clinical diagnoses, counting true positives (TP), false positives (FP), true negatives (TN), and false negatives (FN). Performance metrics were calculated: Sensitivity (TP / (TP +FN)), Specificity (TN / (TN +FP)), Positive Predictive Value (PPV) (TP / (TP +FP)), and Negative Predictive Value (NPV) (TN / (TN +FN)).

#### Receiver operating characteristic (ROC) curve and area under the curve (AUC) value

The pROC package in R was used to compute true positive and false positive rates at different thresholds, and the AUC function was used to calculate AUC values. ROC curves were plotted with AUC values annotated to quantify diagnostic performance.

#### Cohen’s kappa coefficient

The irr package in R was used to calculate Cohen’s kappa coefficient, assessing the consistency between detection methods and clinical diagnoses (kappa interpretation: <0.20: slight; 0.21–0.40: fair; 0.41–0.60: moderate; 0.61–0.80: substantial; 0.81–1.00: almost perfect).

All statistical analyses were performed in R 4.4.0, using packages including dplyr, ggplot2, pROC, and irr. Additionally, IBM SPSS Statistics 25 was used for descriptive statistical analysis, and the χ test or Fisher’s exact test was applied to assess significant differences. A *P*-value < 0.05 was considered statistically significant.

## RESULTS

### Baseline characteristics of clinical participants

All biosamples in this study were obtained from patients at the Senior Department of Tuberculosis, the Eighth Medical Center of PLA General Hospital between December 2020 and April 2023. Detailed information is provided in Table 1.

**TABLE 1:** Patient baseline characteristics

Characteristic	Value				
**Age (years**)	Mean ± SD: 43.56 ± 18.45, IQR: 27.0–58.0				
**Gender (n**)	Female: 77, Male: 56				
**Sample Type (n**)	**MTB**^*a*^**(n**)	**NTM**^*b*^(**n**)	**Mixed**^*c*^**(n**)	**Negative** ^*d*^**(n**)	**Total(n**)
Bronchoalveolar lavage fluid	50	7	5	4	66
Sputum	23	3	6	1	33
Tissue	11	2	0	1	14
Pus	6	2	0	1	9
Cerebrospinal fluid	2	2	1	1	6
Pleural fluid	4	0	0	0	4
Urine	1	0	0	0	1
Total (n)	97	16	12	8	133

^*a*^ MTB: Clinical diagnosis of PTB or EPTB.

^*b*^ NTM: Clinical diagnosis of NTM pulmonary disease.

^*c*^ Mixed: Clinical diagnosis of NTM pulmonary disease (multiple species) and/or PTB.

^*d*^ Negative:Clinical diagnosis includes bacterial meningitis (one case), pulmonary adenocarcinoma (one case), and other cases without a definitive diagnosis.

### Comparison between Ziehl–Neelsen acid-fast staining and nucleic acid MALDI-TOF MS

In the comparative analysis of various sample types, Ziehl–Neelsen acid-fast staining identified 39 positive cases out of 133, yielding a positive detection rate of 39/133 (29.32%). The clinical diagnoses for these 39 acid-fast staining positive samples were as follows: 28 cases of MTB, 6 cases of NTM lung disease, and 5 cases of co-infection with MTB and NTM. Conversely, among the 94 acid-fast staining negative samples, the clinical diagnoses comprised 69 cases of MTB, 10 cases of NTM lung disease, 7 cases of MTB and NTM co-infection, 6 cases with indeterminate diagnoses, 1 case of tuberculous meningitis, and 1 case of lung adenocarcinoma. Sample types are listed in Table 2.

**TABLE 2:** Comparison of *Mycobacterium* detection rates by AFB smear and nucleic acid MALDI-TOF MS among different sample types

Sample type	AFB smear (+)	AFB smear (‒)	MALDI-TOF MS (+)	MALDI-TOF MS (‒)	P
BALF	20 (20/66, 30.3%)	46 (46/66, 69.7%)	58 (58/66, 87.88%)	8 (8/66, 12.12%)	<0.001
Sputum	15 (15/33, 45.45%)	18 (18/33, 54.55%)	30 (30/33, 90.91%)	3 (3/33, 9.09%)	<0.001
Tissue	1 (1/14, 7.14%)	13 (13/14, 92.86%)	11 (11/14, 78.57%)	3 (3/14, 21.43%)	<0.001
Pus	2 (2/9, 22.22%)	7 (7/9, 77.78%)	7 (7/9, 77.78%)	2 (2/9, 22.22%)	>0.056
Pleural fluid	1 (1/4, 25%)	3 (3/4, 75%)	4 (4/4, 100.00%)	0 (0/4, 0.00%)	>0.142
Cerebrospinal fluid	0 (0/6, 0.00%)	6 (6/6, 100%)	3 (3/6, 50.00%)	3 (3/6, 50.00%)	>0.181
Urine	0 (0/1, 0.00%)	1 (1/1,100%)	0 (0/1, 0.00%)	1 (1/1,100%)	1
Total	39	94	113	20	<0.001

Nucleic acid MALDI-TOF MS demonstrated a superior positive detection rate, identifying 113 positive cases out of 113/133 (84.96%). The clinical diagnoses for the 113 nucleic acid MALDI-TOF MS positive samples included 85 cases of MTB, 16 cases of NTM, and 12 cases of MTB/NTM co-infection. Among the 20 nucleic acid MALDI-TOF MS negative cases, the clinical diagnoses included 12 cases of MTB, 6 cases with indeterminate diagnoses, 1 case of tuberculous meningitis, and 1 case of lung adenocarcinoma; no NTM was detected in these negative cases. The sample types are listed in Table 2. Among the 12 samples clinically diagnosed as MTB but negative in nucleic acid MALDI-TOF MS, the distribution is as follows: four cases of BALF, two cases of sputum, two cases of tissue, one case of pus, two cases of cerebrospinal fluid, and one case of urine.

### Evaluation of the diagnostic performance of four detection methods for MTB and NTM

This study assessed the diagnostic performance of four detection methods—nucleic acid MALDI-TOF MS, LJ culture, GeneXpert, and real-time PCR—against clinical diagnoses for MTB and NTM, evaluating sensitivity, specificity, positive predictive value (PPV), and negative predictive value (NPV). The findings are presented in Table 3.

**TABLE 3:** Diagnostic performance of four detection methods for MTB and NTM compared with clinical diagnosis

Detection method	Target	Sensitivity	Specificity	PPV	NPV
MALDI-TOF MS	MTB	88.07% (96/109) [95% CI: 80.66%–92.90%]	95.83% (23/24) [95% CI: 79.76%–99.26%]	98.97% (96/97) [95% CI: 94.39%–99.82%]	63.89% (23/36) [95% CI: 47.58%–77.52%]
	NTM	89.29% (25/28) [95% CI: 72.80%–96.29%]	97.14% (102/105) [95% CI: 91.93%–99.02%]	89.29% (25/28) [95% CI: 72.80%–96.29%]	97.14% (102/105) [95% CI: 91.93%–99.02%]
LJ Culture	MTB	38.53% (42/109) [95% CI: 29.93%–47.91%]	100.00% (24/24) [95% CI: 86.20%–100.00%]	100.00% (42/42) [95% CI: 91.62%–100.00%]	26.37% (24/91) [95% CI: 18.41%–36.25%]
	NTM	42.86% (12/28) [95% CI: 26.51%–60.93%]	100.00% (105/105) [95% CI: 96.47%–100.00%]	100.00% (12/12) [95% CI: 75.75%–100.00%]	86.78% (105/121) [95% CI: 79.60%–91.69%]
Real-time PCR	MTB	40.37% (44/109) [95% CI: 31.64%–49.75%]	95.83% (23/24) [95% CI: 79.76%–99.26%]	97.78% (44/45) [95% CI: 88.43%–99.61%]	26.14% (23/88) [95% CI: 18.09%–36.18%]
	NTM	39.29% (11/28) [95% CI: 23.57%–57.59%]	99.05% (104/105) [95% CI: 94.80%–99.83%]	91.67% (11/12) [95% CI: 64.61%–98.51%]	85.95% (104/121) [95% CI: 78.65%–91.04%]
Gene-Xpert	MTB	60.55% (66/109) [95% CI: 51.17%–69.22%]	100.00% (24/24) [95% CI: 86.20%–100.00%]	100.00% (66/66) [95% CI: 94.50%–100.00%]	35.82% (24/67) [95% CI: 25.40%–47.78%]

Nucleic acid MALDI-TOF MS demonstrated high sensitivity and specificity for both MTB and NTM. For MTB, the sensitivity was 88.07%, and specificity was 95.83%, with PPV and NPV at 98.97% and 63.89%, respectively. In the case of NTM, sensitivity reached 89.29%, specificity was 97.14%, with both PPV and NPV at 89.29% and 97.14%, respectively. This method aligned with clinical diagnoses in 119 out of 133 samples, yielding an overall agreement of 89.47% and a Cohen’s kappa coefficient of 0.702.

LJ culture exhibited a sensitivity for MTB of 38.53% and perfect specificity of 100%, with PPV and NPV at 100% and 26.37%, respectively. For NTM, sensitivity was 42.86%, specificity remained at 100%, with PPV and NPV at 100% and 86.78%, respectively. This method was consistent with clinical diagnoses in 66 out of 133 samples, resulting in an overall agreement of 49.62% and a Cohen’s kappa coefficient of 0.185.

Real-time PCR presented a sensitivity for MTB of 40.37% and specificity of 95.83%, with PPV and NPV at 97.78% and 26.14%, respectively. For NTM, sensitivity was 39.29%, specificity was 99.05%, with PPV and NPV at 91.67% and 85.95%, respectively. This method was consistent with clinical diagnoses in 67 out of 133 samples, achieving an overall agreement of 50.38% and a Cohen’s kappa coefficient of 0.177.

GeneXpert showed a sensitivity for MTB of 60.55% and perfect specificity of 100.00%, with both PPV and NPV at 100.00% and 35.82%, respectively. This method was consistent with clinical diagnoses in 90 out of 133 samples, achieving an overall agreement of 67.67% and a Cohen’s Kappa coefficient of 0.356.

**Fig 2:**
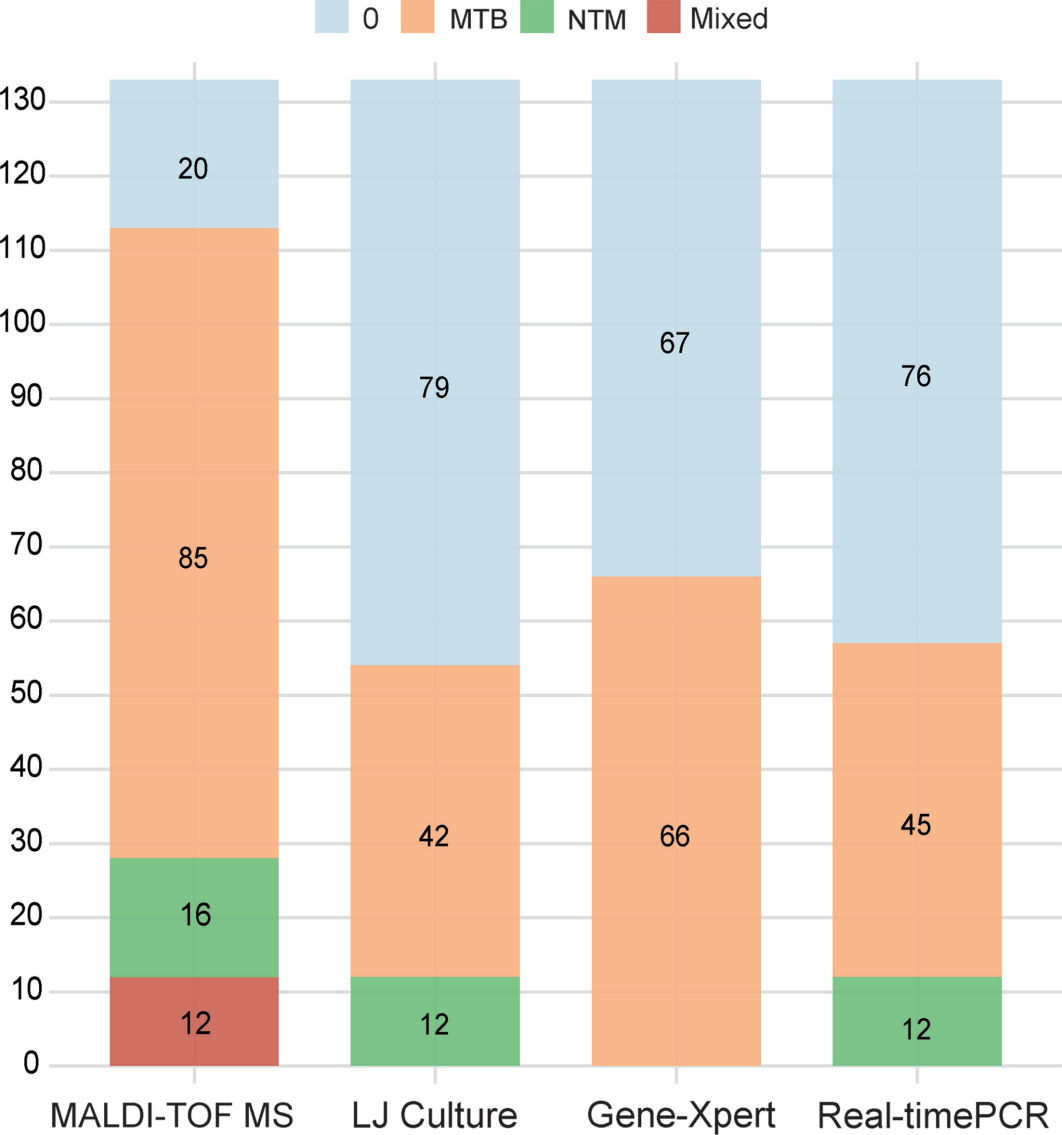
Stacked bar chart displaying the diagnostic results of nucleic acid MALDI-TOF MS, LJ culture, GeneXpert, and real-time PCR. This figure compares the diagnostic performance of the four methods in detecting MTB, NTM, mixed infections, and negative cases. Each bar represents the distribution of diagnostic results across methods, showing the number of MTB, NTM, mixed infections, and negative cases detected by each technique.

The results from different diagnostic methods are summarized as follows: nucleic acid MALDI-TOF MS identified 85 cases of MTB, 16 cases of NTM, 12 cases of MTB/NTM co-infection, and 20 negative cases; LJ culture identified 42 cases of MTB, 12 cases of NTM, and 79 negative cases; GeneXpert identified 66 cases of MTB and 67 negative cases; real-time PCR identified 45 cases of MTB, 12 cases of NTM, and 76 negative cases (Fig. 2). The consistency between LJ culture and nucleic acid MALDI-TOF MS was good, with no cases of LJ culture being positive, while nucleic acid MALDI-TOF MS was negative. However, one case tested negative by nucleic acid MALDI-TOF MS, but both GeneXpert and real-time PCR detected MTB.

### ROC curve analysis of the four detection methods

**Fig 3:**
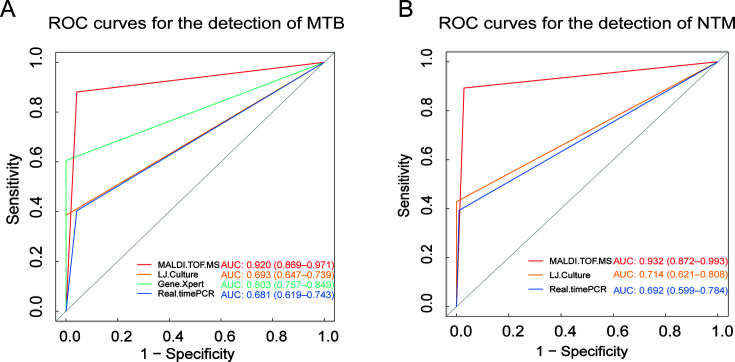
ROC curves for the detection of MTB and NTM using nucleic acid MALDI-TOF MS, LJ culture, the Gene-Xpert assay, and Real-time PCR. Figure 2A delineates the ROC curves for the identification of MTB, highlighting the comparative diagnostic efficacy of nucleic acid MALDI-TOF MS, LJ culture, GeneXpert assay, and real-time PCR. Each curve represents the sensitivity and specificity trade-off at various threshold settings, providing a visual assessment of the diagnostic accuracy for MTB. Figure 2B complements this analysis by presenting the ROC curves for NTM detection, mirroring the methodology applied in Fig. 2A but tailored to the specificities of NTM identification. The curves in Fig. 2B underscore the performance of each diagnostic approach in distinguishing NTM from other mycobacterial species.

The ROC curves for the detection performance of MTB by nucleic acid MALDI-TOF MS, LJ culture, GeneXpert, and real-time PCR are depicted in Fig. 3A. The corresponding AUC values, which indicate the diagnostic accuracy, are as follows: nucleic acid MALDI-TOF MS with an AUC of 0.920 (95% CI: 0.869–0.971), LJ culture with an AUC of 0.693 (95% CI: 0.647–0.739), GeneXpert with an AUC of 0.803 (95% CI: 0.757–0.849), and real-time PCR with an AUC of 0.681 (95% CI: 0.619–0.743). Nucleic acid MALDI-TOF MS exhibited the highest diagnostic performance for MTB, followed by GeneXpert and LJ culture, while real-time PCR showed relatively lower performance.

For NTM detection performance, the ROC curves for nucleic acid MALDI-TOF MS, LJ culture, and real-time PCR are presented in Fig. 3B. The AUC values for these methods are as follows: nucleic acid MALDI-TOF MS with an AUC of 0.932 (95% CI: 0.872–0.993), LJ culture with an AUC of 0.714 (95% CI: 0.621–0.808), and real-time PCR with an AUC of 0.692 (95% CI: 0.599–0.784). Nucleic acid MALDI-TOF MS demonstrated superior diagnostic performance for NTM, followed by LJ culture, with real-time PCR exhibiting the lowest performance among the three methods.

### Diagnostic performance of nucleic acid MALDI-TOF MS in conjunction with other diagnostic modalities for MTB and NTM identification

**Fig 4:**
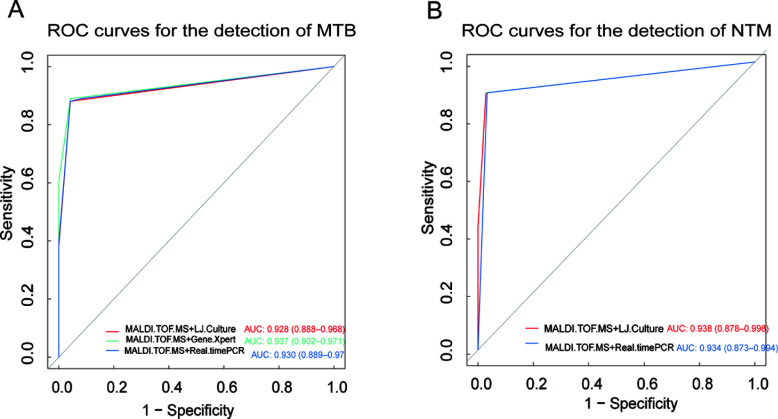
ROC curves for the detection of MTB and NTM using nucleic acid MALDI-TOF MS in conjunction with various diagnostic methods. Figure 3A illustrates the ROC curves for the detection of MTB, showcasing the diagnostic efficacy of nucleic acid MALDI-TOF MS when combined with LJ culture, Gene-Xpert assay, and real-time PCR. Figure 3B presents the corresponding ROC curves for the detection of NTM, demonstrating the performance of the same combined diagnostic approaches.

The synergistic application of nucleic acid MALDI-TOF MS with different diagnostic methods has yielded significant results for the identification of both MTB and NTM. In the case of MTB, the combination with LJ culture resulted in an AUC of 0.928 (95% CI: 0.888–0.968), indicating very good diagnostic performance. This is further corroborated by the AUCs obtained when nucleic acid MALDI-TOF MS was combined with the GeneXpert assay and real-time PCR, achieving AUCs of 0.937 (95% CI:0.902–0.971) and 0.930 (95% CI:0.889–0.970), respectively (Fig. 4A). For NTM, the diagnostic performance was similarly impressive, with the combination of nucleic acid MALDI-TOF MS and LJ culture yielding an AUC of 0.938 (95% CI:0.878–0.998). When nucleic acid MALDI-TOF MS was combined with real-time PCR, the AUC was 0.934 (95% CI:0.873–0.994), demonstrating excellent diagnostic capability (Fig. 4B).

### Nucleic acid MALDI-TOF MS identification of mycobacterial species and comparison with real-time PCR and LJ culture

Among the 133 clinical specimens, nucleic acid MALDI-TOF MS detected 16 cases of NTM, 85 cases of MTB, and 12 cases of MTB/NTM co-infection, with a positive detection rate of 84.96%. Real-time PCR detected 12 cases of NTM and 44 cases of MTB, with a positive detection rate of 42.11%. There was a significant difference in the positive detection rates between nucleic acid MALDI-TOF MS and real-time PCR (*P* < 0.01), with the detailed detection results of both methods presented in Table 4.

**TABLE 4:** Comparative analysis of detection results between real-time PCR and nucleic acid MALDI-TOF MS for *Mycobacterium* identification

Real-time PCR (n)	MALDI-TOF MS (n)
NTM (12)	*M. intracellulare* (2)
	*M. marseillense* (2)
	*M. abscessus* (1)
	*M. bolletii* (1)
	*M. toadense* (1)
	*M. abscessus* and *M. bolletii* (1)
	*M. abscessus* and *M. intracellulare* (1)
	MTB and *M. intracellulare* (1) MTB and *M. abscessus* (1) MTB and *M. bolletii* (1)
MTB (44)	MTB and *M. gordonae* (1)
	*M. intracellulare* (1)
	MTB (42)
Negative (77)	MTB and *M. intracellulare* (1) MTB and *M. kansasii* (1) MTB and *M. minor* (1) MTB and *M. branderi* (1) MTB and *M. ulcerans* (1) MTB and *M. gordonae* (1)
	*M. abscessus* (2)
	*M. kansasii* (1)
	*M. minor* (2)
	*M. intracellulare* (3)
	MTB (43)
	Negative (20)
Total (133)	Total (133)

In addition, the LJ culture method identified 12 cases of NTM and 42 cases of MTB, with a positive detection rate of 40.6%. There was a significant difference in the positive detection rates between nucleic acid MALDI-TOF MS and LJ culture (*P* < 0.01), with the detailed detection results of both methods shown in Table 5.

**TABLE 5:** Comparative analysis of detection results between Löwenstein–Jensen (LJ) culture and nucleic acid MALDI-TOF MS for *Mycobacterium* identification

LJ culture (n)	MALDI-TOF MS (n)
NTM (12)	*M. intracellulare* (2)
	*M. marseillense* (2)
	*M. abscessus* (2)
	*M. kansasii* (1)
	*M. bolletii* (1)
	*M. toadense* (1)
	MTB and *M. minor* (1) *M. abscessus* and *M. bolletii* (1) *M. abscessus* and *M. intracellulare* (1)
MTB (42)	MTB and *M. kansasii* (1)
	MTB and *M. gordonae* (1)
	MTB and *M. bolletii* (1)
	MTB and *M. abscessus* (1)
	MTB (38)
Negative (79)	MTB and *M. intracellulare* (2) MTB and *M. branderi* (1) MTB and *M. gordonae* (1) MTB and *M. ulcerans* (1)
	*M. abscessus* (1)
	*M. minor* (2)
	*M. intracellulare* (4)
	MTB (47)
	Negative (20)
Total (133)	Total (133)

### Comparison of drug resistance detection for 10 anti-tuberculosis drugs by nucleic acid MALDI-TOF MS and traditional DST

Comparative analysis of traditional drug sensitivity and nucleic acid MALDI-TOF MS molecular drug sensitivity results for 40 cases of MTBC is shown in Table 6. The specific consistency rates of nucleic acid MALDI-TOF MS and phenotype DST in drug resistance detection were as follows: INH: 85%, RFP: 95%, EMB: 85%, SM: 90%, LFX: 80%, KM: 82.5%, AM: 85%, CAP: 85%, Pto: 92.5%, and PAS: 85%.

**TABLE 6:** Phenotypic drug sensitivity and MALDI-TOF MS molecular drug sensitivity results for *Mycobacterium tuberculosis* complex

Drug	Phenotypic resistance	Genotypic detection (MALDI-TOF MS)	Consistency rate
Isoniazid (INH)	Low resistance (16)	katG315 AGC-ACC (14) inhA-15 C-T (2)	34/40 (85%)
	High resistance (1)	No mutation detected (1)	
	Sensitive (23)	katG315 AGC-ACC (3) katG316 GGC-AGC (1) inhA-15 C-T (1) No mutation detected (18)	
Rifampicin (RFP)	Low resistance (5)	rpoB531 TCG-TTG (2) rpoB516 GAC-GTC (1) rpoB526 CAC-TAC (1) rpoB511 CTG-CCG (1)	38/40 (95%)
	High resistance (15)	rpoB531 TCG-TTG (12) rpoB516 GAC-GTC (1) rpoB526 CAC-TAC (2)	
	Sensitive (20)	rpoB531 TCG-TTG (1) rpoB533 CTG-CCG (1) No mutation detected (18)	
Ethambutol (EMB)	Low resistance (8)	embB306 ATG-GTG (3) embB306 ATG-ATC (1) No mutation detected (4)	34/40 (85%)
	High resistance (0)	No mutation detected (0)	
	Sensitive (32)	embB306 ATG-GTG (1) No mutation detected (31)	
Streptomycin (SM)	Low resistance (4)	No mutation detected (4)	36/40 (90%)
	High resistance (13)	rpsL43 AAG-AGG (11) No mutation detected (2)	
	Sensitive (23)	No mutation detected (23)	
Levofloxacin (LFX)	Low resistance (8)	gyrA90 GCG-GTG (0) gyrA94 GAC-GCC (1) gyrA94 GAC-GGC (5) No mutation detected (2)	32/40 (80%)
	High resistance (5)	gyrA90 GCG-GTG (1) gyrA90 GCG-GTG +gyrA94 GAC-GGC (1) No mutation detected (3)	
	Sensitive (27)	gyrA94 GAC-GGC (1) gyrA94 GAC-GTC (2) No mutation detected (24)	
Kanamycin (KM)	Low resistance (1)	No mutation detected (1)	33/40 (82.5%)
	High resistance (2)	rrs1401 A-G (2)	
	Sensitive (37)	rrs1484 G-T (6) No mutation detected (31)	
Amikacin (AM)	Low resistance (0)	No mutation detected (0)	34/40 (85%)
	High resistance (2)	rrs1484 G-T (1) rrs1401 A-G (1)	
	Sensitive (38)	rrs1484 G-T (6) No mutation detected (32)	
Capreomycin (CAP)	High resistance (1)	rrs1484 G-T (1)	34/40 (85%)
	Sensitive (39)	rrs1484 G-T (5) rrs1401 A-G (1) No mutation detected (33)	
Prothionamide (Pto)	High resistance (1)	inhA-15 C-T (1)	37/40 (92.5%)
	Sensitive (39)	inhA-15 C-T (3) No mutation detected (36)	
Para-aminosalicylic acid (PAS)	Low resistance (4)	No mutation detected (4)	34/40 (85%)
	High resistance (2)	No mutation detected (2)	
	Sensitive (34)	No mutation detected (34)	

### Comparison of rifampicin resistance detection between nucleic acid MALDI-TOF MS and Xpert MTB/RIF

A comparative analysis of rifampicin resistance detection was conducted between nucleic acid MALDI-TOF MS and Xpert MTB/RIF across 57 positive samples with molecular drug susceptibility results. Xpert MTB/RIF identified 25 cases as rifampicin-sensitive, yet nucleic acid MALDI-TOF MS detected an rpoB522TCG-TCT mutation in one instance, which is a synonymous mutation encoding serine. The remaining 24 cases lacked any genotypes associated with rifampicin resistance. Among the 32 rifampicin-resistant cases identified by Xpert MTB/RIF, nucleic acid MALDI-TOF MS revealed various mutations: 18 cases exhibited rpoB531TCG-TTG, four cases showed rpoB511CTG-CCG, two cases had rpoB526CAC-CTC, and single cases displayed rpoB526CAC-TAC, rpoB526CAC-AAC, rpoB533CTG-CCG, and rpoB516GAC-GTC, and one case presented dual mutations of rpoB531TCG-TTG and rpoB526CAC-TAC. Notably, in three cases, no rifampicin resistance genotypes were detected by nucleic acid MALDI-TOF MS, resulting in a rifampicin resistance detection consistency rate of 94.74% (Table 7).

**TABLE 7:** Molecular drug susceptibility results of Xpert MTB/RIF and MALDI-TOF MS

Xpert MTB/RIF	MALDI-TOF MS	Detection consistency
Rifampicin-resistant (32)	rpoB531TCG-TTG (18)	54/57 (94.74%)
	rpoB511CTG-CCG (4)	
	rpoB526CAC-CTC (2)	
	rpoB526CAC-TAC (1)	
	rpoB526CAC-AAC (1)	
	rpoB533CTG-CCG (1)	
	rpoB516GAC-GTC (1)	
	rpoB531TCG-TTG + rpoB526CAC-TAC (1)	
	No mutation detected (3)	
Rifampicin-sensitive (25)	rpoB522TCG-TCT (1)^a^	
	No mutation detected (24)	

^a^ synonymous mutation.

### Comparison of rifampicin resistance detection among nucleic acid MALDI-TOF MS, GeneXpert, and traditional drug susceptibility testing

An analysis was conducted on 29 positive samples that had simultaneously collected traditional culture drug susceptibility results, GeneXpert, and nucleic acid MALDI-TOF MS molecular drug susceptibility results. Among the 17 cases that were rifampicin-resistant according to traditional drug susceptibility testing, GeneXpert results indicated resistance in all cases. Nucleic acid MALDI-TOF MS results showed rpoB531TCG-TTG mutation in 11 cases, rpoB526CAC-CTC mutation in two cases, rpoB516GAC-GTC mutation in one case, a combination of rpoB531TCG-TTG and rpoB526CAC-TAC mutations in one case, and no rifampicin resistance genotypes detected in two cases. Among the 12 cases identified as rifampicin-sensitive by DST, nine cases did not show resistance in the Gene-Xpert test, and MALDI-TOF MS also did not detect rifampicin resistance-related mutations in these nine cases. However, in the three cases where GeneXpert detected resistance despite DST indicating rifampicin sensitivity, nucleic acid MALDI-TOF MS identified specific resistance mutations: one case with the rpoB531TCG-TTG mutation, one case with the rpoB511CTG-CCG mutation, and one case with the rpoB533CTG-CCG mutation.

## DISCUSSION

TB stands as a persistent global health challenge, with the rise of MDR and extensively drug-resistant (XDR) TB strains intensifying the threat to public health (35–38). The increasing prevalence of NTM infections worldwide adds another layer of complexity to the diagnosis and management of mycobacterial diseases (10, 11, 39). Timely and accurate differentiation between TB and NTM, along with the assessment of their drug resistance profiles, is crucial for controlling the spread of drug-resistant strains, enhancing treatment efficacy, and reducing mortality rates, thereby alleviating the economic burden on affected individuals.

The advent of molecular diagnostics has marked a significant shift from traditional culture-based methods, which, despite their reliability, are constrained by lengthy turnaround times that impede rapid clinical intervention (40, 41). The GeneXpert MTB/RIF assay, a molecular method that detects MTB and rifampicin resistance, has made strides in expediting diagnostic processes but has limitations in its comprehensive coverage of resistance genes (26, 28). Isothermal amplification techniques are convenient to operate, but they can only detect MTB and cannot provide information on drug resistance (42). Gene chips and linear probe techniques can identify bacterial species and multiple drug resistance-related gene loci with high sensitivity and specificity, but their detection range is limited, and the hybridization and washing steps are cumbersome (43–45). Genetic sequencing technologies offer a broad detection range for various pathogens and drug resistance mutations but demand rigorous sample, equipment, and interpretive standards, with first-generation sequencing facing limitations in detecting heteroresistance (42). Consequently, there is an unmet need for diagnostic technologies that can rapidly and accurately identify MTB, NTM, and their respective drug resistances, thereby facilitating the development of early and personalized treatment plans and ultimately reducing the prevalence and impact of TB.

Nucleic acid MALDI-TOF MS technology integrates matrix-assisted laser desorption/ionization with time-of-flight mass spectrometry, enabling rapid sample analysis and identification within a short timeframe (30 min to 1 h) (43). This technology excels in the rapid identification of MTB and NTM, with a high positive detection rate and the capability to accurately detect resistance patterns (44). Nucleic acid MALDI-TOF MS is designed to detect conserved gene fragments of mycobacteria, such as 16S rRNA and hsp65, facilitating the rapid and accurate identification of various mycobacterial species. Moreover, it can identify gene polymorphisms associated with drug resistance, such as those in the *rpoB*, *katG*, *gyrA*, and *erm(41*) genes, providing valuable information on resistance gene mutations (43, 45). Compared with traditional nucleic acid amplification techniques that target a limited number of gene loci, nucleic acid MALDI-TOF MS can detect polymorphisms in a broader range of gene loci, such as the ITS region and *rpoB* gene, significantly enhancing the sensitivity and specificity of diagnostics (45). The technology can identify eight subspecies of the MTB complex and 40 NTM species and subspecies, and its application in resistance detection covers most common anti-TB and anti-NTM drugs, including first-line and second-line anti-TB drugs, as well as macrolide and aminoglycoside resistance genotypes in NTM. Nucleic acid MALDI-TOF MS can also detect heteroresistance and synonymous mutations, offering a high-throughput, rapid, accurate, sensitive, high-resolution, and cost-effective diagnostic solution that is independent of fluorescent probes and similar reagents, with a shorter detection cycle than first- and second-generation sequencing.

Our study compared MALDI-TOF MS with traditional phenotypic methods and molecular diagnostic methods, including acid-fast staining, LJ culture, GeneXpert, and real-time PCR. The results showed that acid-fast staining detected 39 positive cases, with a positive detection rate of 29.32%, while nucleic acid MALDI-TOF MS detected 113 positive cases, achieving a significantly higher positive detection rate of 84.96%. MALDI-TOF MS demonstrated a higher positive detection rate than acid-fast staining across different sample types, particularly in BALF, sputum, and tissue samples (*P* < 0.001), indicating significantly improved diagnostic efficiency. Nucleic acid MALDI-TOF MS can serve as an effective tool for mycobacterial detection, especially for AFB-negative samples. The sensitivity of nucleic acid MALDI-TOF MS for MTB detection was significantly higher than other methods, with a specificity of 95.83%, indicating its diagnostic accuracy and reliability. ROC curve analysis further confirmed the excellent diagnostic capability of MALDI-TOF MS, with an AUC of 0.92, outperforming LJ culture (AUC = 0.693), GeneXpert (AUC = 0.803), and real-time PCR (AUC = 0.681). For NTM detection, the sensitivity and specificity of MALDI-TOF MS were 89.29% and 97.14%, respectively, with an AUC of 0.932, exceeding LJ culture (AUC = 0.714) and real-time PCR (AUC = 0.692). Cohen’s kappa coefficient further demonstrated the high consistency between MALDI-TOF MS and clinical diagnosis, with an agreement rate of 89.47% and a coefficient of 0.702, significantly better than LJ culture (0.185), GeneXpert (0.356), and real-time PCR (0.177). The findings of our study are corroborated by recent research. Baiying Li et al. reported high sensitivity, specificity, and accuracy of nucleic acid MALDI-TOF MS in mycobacterial identification, with values reaching 96.91%, 100%, and 97.22%, respectively (46). Zhu et al. found an overall correct detection rate of 91.64% for clinical mycobacterial isolates using nucleic acid MALDI-TOF MS, identifying mixed infections in 18.65% of the cases (47). Yao et al. reported sensitivities and specificities for NTM detection at 77.8% and 92.5%, respectively (48). Our study’s results, along with these reports, underscore the potential of nucleic acid MALDI-TOF MS in rapidly distinguishing TB from NTM disease and providing a comprehensive resistance profile.

In terms of mycobacterial species identification, our study detected 28 cases of NTM among 133 clinical samples, including *M. intracellulare* and *M. abscessus* complex, aligning with the increasing annual incidence of NTM infections and the predominance of these pathogens (11, 16). Furthermore, nucleic acid MALDI-TOF MS identified mixed mycobacterial infections in 12 cases, a capability that surpasses the scope of real-time PCR, which can only differentiate between MTB complex and NTM without species-level identification. However, one case yielded a negative result with nucleic acid MALDI-TOF MS, while both GeneXpert and real-time PCR detected MTB as positive. Further investigation identified this case as a pulmonary tuberculosis patient, with the sample being BALF. Possible reasons for the inconsistency include a low bacterial load in the sample, which may not have been adequately mixed during aliquoting, leading to uneven bacterial distribution, differences in the target genes analyzed by the various diagnostic methods, and the potential for false-negative results in nucleic acid MALDI-TOF MS detection.

For drug resistance detection in MTB, our comparative analysis between traditional phenotypic drug susceptibility testing and nucleic acid MALDI-TOF MS molecular drug susceptibility testing revealed high consistency rates for isoniazid, rifampicin, ethambutol, streptomycin, levofloxacin, kanamycin, amikacin, capreomycin, prothionamide, and p-aminosalicylic acid resistance detection, with rates above 90% for rifampicin, streptomycin, and prothionamide. This aligns with recent findings indicating a consistency rate between nucleic acid MALDI-TOF MS and phenotypic sensitivity results for various drugs, ranging from 81.8% to 93.9% (49). However, we have also observed some inconsistent results, such as the presence of drug resistance mutations in strains that are phenotypically susceptible. These inconsistencies may be due to the high sensitivity of nucleic acid MALDI-TOF MS technology in detecting heterogeneous resistance, allowing it to identify even a very small proportion of resistant strains. In contrast, GeneXpert and phenotypic drug susceptibility tests are unable to detect low proportions of resistant strains.

In the case of isoniazid phenotypic resistance, nucleic acid MALDI-TOF MS detected katG315 AGC-ACC mutations in 14 out of 16 cases, all of which were low-level INH resistance, suggesting that high-dose isoniazid treatment could be considered when the molecular drug susceptibility report shows katG315 AGC-ACC mutations. In addition, nucleic acid MALDI-TOF MS identified the inhA-15C-T mutation, associated with isoniazid resistance, in six cases. This mutation site is also relevant for resistance to ethionamide and prothionamide, suggesting that when the inhA-15C-T mutation is detected, these drugs should generally be avoided as treatment options. This finding has significant clinical implications, especially for primary healthcare institutions that are limited to performing isoniazid drug sensitivity testing.

Heteroresistance is a phenomenon that cannot be ignored. With the development and widespread application of molecular diagnostic technologies, heteroresistance from a genotypic perspective refers to the coexistence of wild-type and various resistant gene sequences within the same sample (50). Increasingly, studies have reported heteroresistance in MTB samples from TB patients, often leading to inconsistencies between genotypic and phenotypic testing results and delayed accurate diagnosis (51–55). Nucleic acid MALDI-TOF MS technology has high sensitivity for detecting heteroresistance, capable of detecting 1% resistant bacteria (56). For example, as shown in Table 6, genotypic mutations were detected in phenotypically sensitive samples, indicating possible heteroresistance, such as in 5 out of 23 phenotypically sensitive INH cases detected with genotypic mutations by nucleic acid MALDI-TOF MS. This suggests the coexistence of INH-resistant and INH-sensitive strains within the same sample, highlighting the need for clinicians to pay close attention to heteroresistance when formulating treatment plans.

In the 57 positive samples with simultaneously collected GeneXpert and nucleic acid MALDI-TOF MS molecular drug susceptibility testing results (Table 7), GeneXpert detected 25 cases as rifampicin-sensitive. Among these, nucleic acid MALDI-TOF MS detected an rpoB522TCG-TCT synonymous mutation in one case, which encodes serine. A synonymous mutation refers to a gene mutation that does not result in a change in the encoded amino acid and does not lead to drug resistance. Common synonymous mutations include the codon 513 mutation of the rpoB gene from CAA to CAG, both encoding glutamine; the codon 533 mutation of the rpoB gene from CTG to TTG, both encoding leucine; and the codon 90 mutation of the gyrA gene from GCG to GCA, both encoding alanine. These mutations do not alter the target proteins of rifampicin and quinolones; they do not induce resistance, allowing for continued use of rifampicin and quinolones in clinical treatment.

GeneXpert identified 32 cases as rifampicin-resistant, whereas nucleic acid MALDI-TOF MS failed to detect resistance genotypes in three cases. The concordance rate for rifampicin resistance detection between the two methods was 94.74% (54/57). Potential reasons for the discordant results include (i) low abundance of MTB: low MTB counts in the samples may have impeded the detection capability of nucleic acid MALDI-TOF MS, leading to inconsistencies; (ii) limited coverage of known mutations: nucleic acid MALDI-TOF MS relies on a database of known mutation information, which may not encompass all resistance genes and mutation sites. This limitation can result in missed detections. The clinician should comprehensively judge and adjust the drug according to the treatment results.

Additionally, for the comparison of 29 positive samples simultaneously collected for traditional culture drug susceptibility results, GeneXpert, and nucleic acid MALDI-TOF MS molecular drug susceptibility results, the consistency rate for rifampicin resistance detection between traditional culture drug susceptibility and nucleic acid MALDI-TOF MS was 24/29 (82.76%). Three of the phenotypic drug susceptibility tests showed rifampicin sensitivity, while nucleic acid MALDI-TOF MS indicated rifampicin resistance. The reason for this inconsistency may be that nucleic acid MALDI-TOF MS has high sensitivity for detecting a small number (e.g., 1%) of resistant bacteria in the sample, allowing it to detect heteroresistance. The consistency rate between GeneXpert and nucleic acid MALDI-TOF MS was 29/29 (100%). Rifampicin resistance was primarily caused by the rpoB531TCG-TTG mutation (11/17), accounting for 64.71%.

This study has several limitations. First, as a retrospective study, it is unable to effectively address and resolve inconsistencies in the results. Second, not all samples were subjected to all available methods, which complicates the comprehensive comparison between techniques. Third, some positive samples did not undergo drug resistance testing, and NTM were not subjected to drug susceptibility testing, leading to incomplete resistance data. This is partly because most NTM strains exhibited resistance to the 10 drugs tested, and key drugs for the treatment of NTM infections, such as azithromycin, clarithromycin, and rifabutin, were not included in the testing panel. Finally, nucleic acid MALDI-TOF MS relies on known drug resistance genes and mutation sites in the database and cannot encompass all resistance mechanisms or unknown drug resistance mutations. Therefore, traditional culture and phenotypic drug susceptibility testing remain essential, as they can compensate for these limitations.

### Conclusions

Nucleic acid MALDI-TOF MS has demonstrated significant advantages in the rapid and accurate detection of mycobacterial species identification and drug resistance in MTB, applicable to various types of samples. This technology can significantly reduce the detection time, aiding in the early diagnosis of tuberculosis, timely initiation of effective treatment, thereby lowering the risk of transmission and reducing mortality rates. Additionally, compared with phenotypic drug susceptibility testing, nucleic acid MALDI-TOF MS has the advantage of detecting resistance not only to first-line anti-tuberculosis drugs but also to second-line drugs, such as bedaquiline, linezolid, FQs, clofazimine, and cycloserine.

As research into mycobacterial drug resistance genes and loci, particularly those of *Mycobacterium tuberculosis*, continues to advance, the scope of drug resistance detection capabilities of nucleic acid MALDI-TOF MS will also expand to include new resistance genes and loci. To overcome current limitations and enhance the reliability and applicability of results, future studies should aim to broaden the sample size, refine experimental designs, and integrate multiple detection methods to form a more comprehensive diagnostic system. This approach will improve overall diagnostic efficacy and further enhance the accuracy and reliability of tuberculosis diagnostics.

Providing a solid basis for the formulation of personalized treatment plans, nucleic acid MALDI-TOF MS holds significant implications for improving public health conditions in regions with high incidences of tuberculosis. It is imperative to recognize the scientific and public health importance of this technology in advancing our capabilities to combat this persistent disease.

## Data Availability

All data generated or analyzed during this study were included in this published article.
